# Determinants of Sugar-Sweetened Beverage Consumption Among Adults in Perambalur District of India

**DOI:** 10.7759/cureus.35650

**Published:** 2023-03-01

**Authors:** Ezhilnila Subramanian, Tamilarasan Muniyapillai, Maniprabhu S, Karthikeyan Kulothungan, Muthuraaj Kumar R S

**Affiliations:** 1 Physiology, Dhanalakshmi Srinivasan Medical College and Hospital, Perambalur, IND; 2 Community Medicine, Dhanalakshmi Srinivasan Medical College and Hospital, Perambalur, IND; 3 Community Medicine, K.A.P. Viswanatham Medical College, Trichy, IND

**Keywords:** socio-demographic factors, from india, lifestyle behaviours, obesity and overweight, sugar sweetened beverages

## Abstract

Background

Sugar is a ubiquitous element in processed meals and is a major source of the energy we derive from them. The risk of obesity and other chronic conditions, such as high blood pressure, cardiovascular disease, type 2 diabetes mellitus, tooth destruction, as well as dental cavities, increases proportionally with the consumption of sugar-sweetened beverages (SSBs). This study intends to determine the prevalence of SSB intake among adults in the Perambalur area of Tamil Nadu, India, as well as the factors that influence it.

Methodology

We surveyed 1007 individuals using a cross-sectional design from June to November 2022. We included residents who were at least 18 years old and less than 80 years old. Using a convenience sample method, we gathered responses from the public in the urban and rural field practice areas of a teaching medical college in the district of Perambalur, India. We conducted in-person interviews to get data regarding the consumption of SSBs. Among other socio-demographic information, the participants' names, ages, religions, levels of education and employment, household incomes, family compositions, marital statuses, lifestyle behaviors, and comorbid conditions were also gathered. We measured the SSB consumption frequency and duration, and we also considered the contexts in which they consumed SSBs. We examined the factors that play a role in SSB consumption and questioned the participants' familiarity with SSBs' constituents, adverse effects, and cumulative toll. Besides examining the effects of SSB use, the research also explores the possibility of reducing or stopping it altogether.

Results

The prevalence of SSB use among the current study population was 96.3%. Half of the population has consumed SSBs, between 100 and 200 ml, for over 10 years. Taste and peer pressure are the primary reasons for facilitating SSBs, whereas the media has a minor impact. Most of the population (69%) began consuming SSBs, mostly during vacations and at parties. About one-fifth of the population experiences negative consequences after ingesting SSBs, while only half of the population is aware of the contents of SSBs. Likewise, just 50% of the population is aware of the long-term implications of SSBs. Nearly 16.7% of the population attempted to stop using SSBs. Being overweight, belonging to a high socioeconomic class, and dwelling in a rural location are risk factors related to the consumption of SSBs.

Conclusion

The prevalence of SSB use among the current study population is exceptionally high. Being overweight, belonging to a high socioeconomic class, and dwelling in a rural location are risk factors related to the consumption of SSBs. There is a need to educate the public about the short- and long-term negative effects of consuming SSBs. Government and non-government entities must work together to generate public behavior change communication.

## Introduction

Originating in India, sugar is the most extensively consumed sweetener worldwide. The English word "sugar" is derived from the Sanskrit word "sarkara," which signifies gravel [1]. Sugar is a ubiquitous element in many processed meals and is a major source of the energy we derive from them [2]. Particularly among young people, there has been an increase in the consumption of sugary sodas, flavored carbonated water, and juice with added sugar while milk consumption has decreased significantly [3]. This article refers to these beverages collectively as "sugar-sweetened beverages" (SSBs).

SSBs are defined by the World Health Organization, as “all types of beverages containing free sugars and these include carbonated or non-carbonated soft drinks, fruit/vegetable juices and drinks, liquid and powder concentrates, flavored water, energy and sports drinks, ready-to-drink tea, ready-to-drink coffee, and flavored milk drinks” [4].

Since 1998, SSB sales in India have increased by 13% per year, reaching about 11 liters per person per year [5]. Some nations have reduced the prevalence of SSB use by implementing higher tariffs and health warnings on SSB packaging [6]. The World Health Organization currently recommends that both adults and children limit their everyday consumption of free sugars to less than 10% of their total calorie intake [7]. The Food Safety and Standards Authority of India (FSSAI) recommended a reduction in SSB consumption and imposing additional taxes on sugar-sweetened carbonated beverages to reduce the prevalence of non-communicable chronic diseases among the Indian population [8]. In India, people must pay the Goods and Services Tax (GST) on sugary drinks, especially sodas.

The risk of obesity [1,3,9] and other chronic conditions, such as high blood pressure [10-12], cardiovascular disease [10], type 2 diabetes mellitus [5,12,13], tooth destruction, as well as dental cavities [14,15], increases proportionally with SSB consumption.

Consumption of SSBs is associated with age, gender, education, and socioeconomic status, among other demographic and socioeconomic factors [16,17]. People between the ages of 18 and 39 consume the most SSBs, according to research [16-19]. According to most studies, males consume a much higher proportion of SSBs than females do [16,17,20]. Multiple studies have revealed a relationship between lower levels of education and greater rates of SSB usage [16,19,21]. Being of lower socioeconomic class and never married or divorced has also been associated with increased consumption of SSBs [16,20,22]. There is a relationship between greater use of sugary beverages and obesity, as well as other factors, such as watching television for long periods of time and eating a diet that is high in fried foods [23,24].

Most of the research regarding the parameters related to SSB consumption comes from high-income nations or countries that have strict policies in place to regulate the amount of sugary drinks that their citizens consume [2]. The consumption trends of SSBs among various socioeconomic and demographic categories in low- and middle-income countries such as India have only a few studies. There is a shortage of research examining the multitude of factors that influence the intake pattern of SSBs among adult Indians. This study intends to determine the prevalence of SSB intake among adults in the Perambalur area of Tamil Nadu, India, as well as the factors that influence it.

## Materials and methods

Study design

The current research is an analytical cross-sectional study.

Study population, place, and duration

We surveyed individuals in the Perambalur area using a cross-sectional design. The town of Perambalur is in Tamil Nadu, India. It serves as the administrative Centre for the Perambalur district and taluk. The population of the municipality was 49,648 as of the 2011 census. Between June and November 2022, we met with the participants in person for interviews.

Ethics clearance

Before beginning the study, we got approval from the Institutional Ethics Committee on human subjects (approval number: IECHS/IRCHS/No. 206).

Selection criteria

We included residents of the Perambalur district who were at least 18 years old and less than 80 years old.

Sample size and sampling method

According to a study conducted by Barrett et al., 20.4% of people consume SSBs daily [20]. Considering the above prevalence, we calculated the sample size using the formula n = 3.84*p*q/d2 [p = 20.4, q = 79.6, d = 3], and the sample size came up to 683. Using a convenience sample method, we gathered responses from 1,007 members of the public in the urban and rural field practice areas of Teaching Medical College in the district of Perambalur.

Data collection

We devised a proforma and conducted in-person interviews to get data regarding the consumption of SSBs. Among other socio-demographic information, the participants' names, ages, religions, levels of education and employment, household incomes, family compositions, and marital statuses were obtained. We collected data on the lifestyle behaviors and comorbid conditions of the participants.

For analysis, we reclassified the frequency with which participants reported consuming SSBs (daily, weekly, occasionally, or never) into two groups: respondents reporting drinking SSBs (daily, weekly, occasionally, or never) and respondents reporting not drinking SSBs (never).

We tracked how often and for how long people consumed SSBs, and we inquired about the circumstances in which they did so. We examined the factors that play a role in SSB consumption. The study also included questions about participants' familiarity with SSBs' constituents, adverse effects, and cumulative toll. Besides examining the effects of SSB use, the research also explores the possibility of reducing or stopping it altogether.

Statistical analysis

The data were entered into Microsoft Excel (Microsoft Corp., Redmond, WA) and then analyzed with SPSS version 21. (IBM Corp., Armonk, NY). We performed the descriptive analysis and presented the data either as frequencies and percentages or as averages and standard deviations. Depending on the data, we used chi-square analysis or Fisher's exact test to see if there was a link between demographic factors and the frequency with which people consumed SSBs.

## Results

About 1007 participants took part in our study. In Table 1, we detail the typical characteristics of the study participants. The participants' average age was approximately 37 years and their average body mass index (BMI) was 25.9. Most responders (61.7%) were male and lived in urban areas (56.6%). Nearly 62.5% of the participants in the survey were college graduates, and roughly 30% of them were professionals. Most of them were Hindu (84.7%), and they lived in nuclear families (83.6%). The prevalence of smoking, drinking, and betel nut chewing was 11.2%, 19.2%, and 2.4%, respectively, among the subjects. Almost 29% of the patients had at least one known comorbidity from Table 1. The prevalence of hypertension was 15.39% among research participants. Sixty-four percent of the individuals in the study belonged to Class 1 on the socioeconomic status continuum.

**Table 1:** General characteristics of the study participants (n = 1007) *Multiple options The cut-offs for the BMI of Asians are as follows: Obese - more than 27.5 kg/m^2^ Underweight - less than 18.5 kg/m^2^ Overweight - 23 to 27.5 kg/m^2^

S.no	Variables		Frequency	Percent
1	Age in years		Mean–36.94 Standard deviation – 14.55	
2	Number of family members		Mean–4.2 Standard deviation – 1.7	
3	Body mass index		Mean–25.9 Standard deviation – 3.8	
4	Gender	Female	386	38.3
		Male	621	61.7
5	Residence	Rural / Village	437	43.4
		Urban / City	570	56.6
6	Education	Graduate	629	62.5
		Post-graduate	112	11.1
		High school	79	7.8
		Secondary	121	12
		Primary	35	3.5
		No education	31	3.1
7	Occupation	Professional	300	29.8
		Retired	22	2.2
		Office worker	202	20.1
		Self-employed	96	9.5
		Student	134	13.3
		Skilled labor	110	10.9
		Unskilled labor	43	4.3
		Unemployed	100	9.9
8	Religion	Christian	94	9.3
		Hindu	853	84.7
		Muslim	57	5.7
		Others	3	.3
9	Type of family	Three generations	54	5.4
		Joint	111	11.0
		Nuclear	842	83.6
10	Smoking	Yes	113	11.2
		No	894	88.8
11	Alcohol	Yes	193	19.2
		No	814	80.8
12	Betel nut chewing	Yes	24	2.4
		No	983	97.6
13	Any persistent illness^*^	Cardiovascular diseases	33	3.2
		Hypertension	155	15.39
		Diabetes mellitus	114	11.3
		Liver diseases	9	1
		Renal diseases	19	1.8
		GI disorders	76	7.54
		None	712	71
14	BMI category	Underweight	24	2.4
		Normal	365	36.2
		Overweight	618	61.4
15	Socio-economic status according to the Modified P.G. Prasad scale	Class 1	646	64.2
		Class 2	201	20.0
		Class 3	84	8.3
		Class 4	66	6.6
		Class 5	10	1.0

Table 2 describes the characteristics associated with SSB consumption and their distribution across the study participants. The prevalence of SSB consumption among the participants of the study was 96.3%. One-third of the subjects had consumed SSBs between 11 and 20 years. Sixty-five percent of them consumed SSBs occasionally, and around half of them consumed 100 to 200 ml at a time.

**Table 2:** Distribution of the study participants according to variables related to consumption of SSBs (n = 1007) SSBs: sugar-sweetened beverages

S.no	About consumption of SSBs		Frequency	Percent
1	Do you consume sugar-sweetened drinks?	Yes	970	96.3
		No consumption	37	3.7
2	How long have you been drinking SSBs?	No consumption	37	3.7
		Less than 1 year	16	1.6
		1 - 5 years	170	16.9
		6 - 10 years	292	29.0
		11 - 20 years	365	36.2
		More than 20 years	127	12.6
3	How often do you drink SSBs?	No consumption	37	3.7
		Occasionally	608	60.3
		Weekly	337	33.5
		Daily	25	2.5
4	The average amount of drink you consumed at a time?	No consumption	37	3.7
		100 - 200 ml	501	49.7
		200 - 500 ml	443	44.0
		More than 500 ml	26	2.6

In Table 3, we describe the influential factors for initiating the consumption of SSBs among the study participants. Just over two-thirds of the individuals in the study confessed that the flavor of the SSBs influenced them while almost one-third of them reported being influenced by peer pressure.

**Table 3:** Distribution of study participants according to their influential factor for starting the habit of drinking SSBs (n = 970) SSBs: sugar-sweetened beverages

What is your influencing factor to start the habit?		Frequency	Percent
Taste	Yes	721	74.3
	No	249	25.7
Media	Yes	59	6.1
	No	911	93.9
Peer pressure	Yes	336	34.6
	No	634	65.4
None	Yes	36	3.7
	No	934	96.3

Table 4 describes the study participants based on when they consumed SSBs. Most of the survey participants (69%) reported that they drink SSBs throughout any trip or vacation. Second, about 61.4% admitted to consuming SSBs at any party or event.

**Table 4:** Distribution of study participants according to on what occasion they consume SSBs (n = 970) SSBs: sugar-sweetened beverages

On what occasion do you consume SSBs?		Frequency	Percent
Watching TV	Yes	118	12.2
	No	852	87.8
Reading books or newspaper	Yes	23	2.4
	No	947	97.6
Party or treat	Yes	618	63.7
	No	352	36.3
Trip or vacation	Yes	695	71.6
	No	275	28.4
After sports or play	Yes	142	14.6
	No	828	85.4
After meals	Yes	365	37.6
	No	605	62.4
Others	Yes	165	17.0
	No	805	83.0

**Figure 1:**
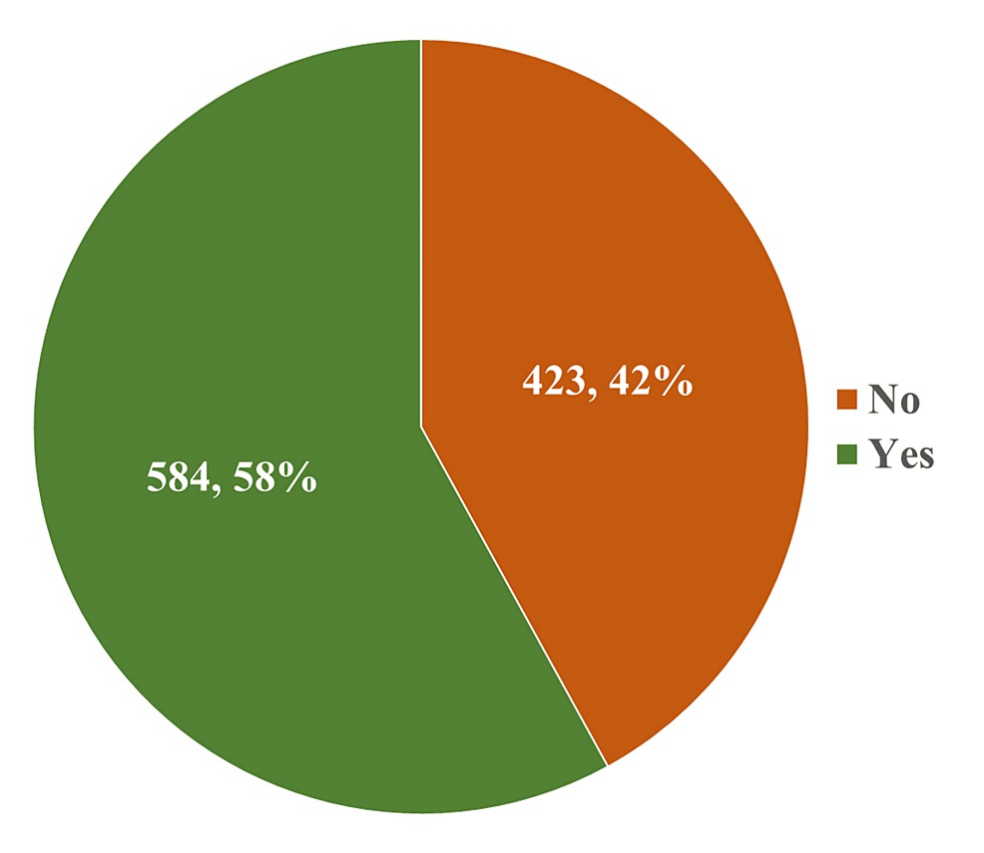
Distribution of the study participants according to their awareness of the ingredients of SSBs (n = 1007) SSBs: sugar-sweetened beverages

We describe the study participants based on their familiarity with the constituents of SSBs (Figure 1). Fifty-eight percent of SSB users were aware of the contents of the product.

**Figure 2:**
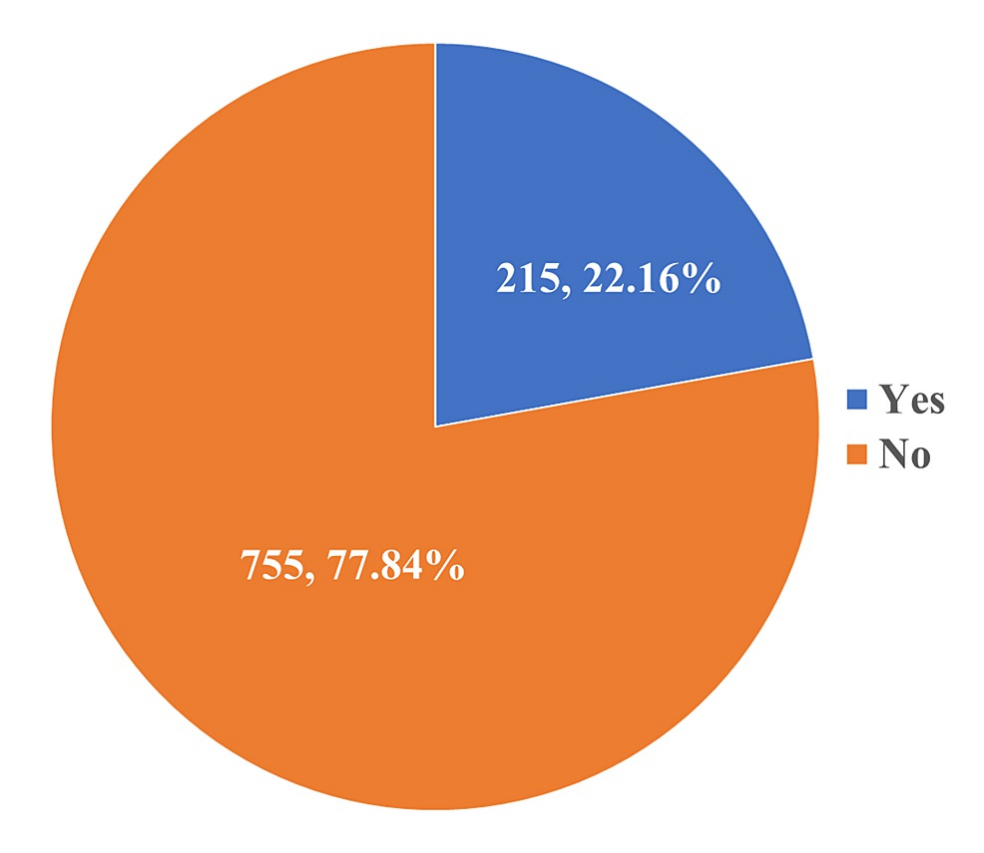
Distribution of the study participants according to their experience of ill effects after consuming SSBs (n = 970) SSBs: sugar-sweetened beverages

Figure 2 describes the study subjects according to the adverse effects they experienced after drinking SSBs. Nearly 22.1% of the participants in the study had experienced adverse effects from SSBs.

Table 5 details the adverse consequences of SSB consumption among the study subjects. After drinking SSBs, almost 97% of the subjects experienced gastrointestinal discomfort, with or without belching. Colds or sinusitis and headaches were the second and third most prevalent side effects.

**Table 5:** Distribution of study participants according to the nature of ill effects after consuming SSBs (n = 215) *Multiple options SSBs: sugar-sweetened beverages

S.no	Mention the ill effects that you experienced after consuming SSBs	Frequency	Percent
1	Gastrointestinal problems with or without belching	210	97
2	Cold or sinusitis	34	16
3	Diarrhea	6	3
4	Oral ulcer	7	4
5	Headache	38	18
6	Dental caries	5	2
7	Disoriented	5	2

**Figure 3:**
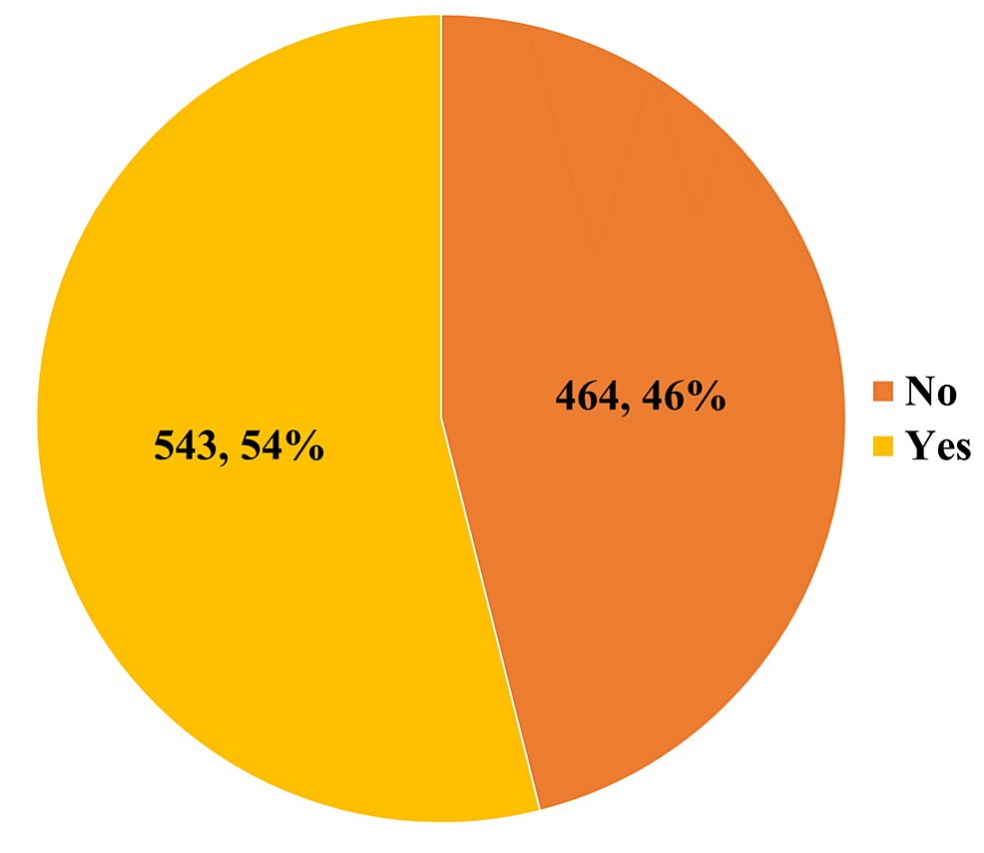
Distribution of study participants according to their awareness of the long-term effects of consuming SSBs SSBs: sugar-sweetened beverages

Figure 3 depicts the proportion of individuals who were aware of the long-term effects of SSB consumption. Approximately 54% of the participants in the study reported being aware of the long-term impacts.

In Table 6, we describe the participants' awareness of the long-term effects of SSB consumption. Sixty percent of the participants in the survey perceived diabetes to be a long-term consequence followed by dental caries (56%), gastritis (48%), and habitual addiction (47%).

**Table 6:** Distribution of study participants according to the awareness of the long-term effects of drinking SSBs (n = 543) *Multiple options SSBs: sugar-sweetened beverages

S.no	Mention the long-term effects you are aware of after consuming SSBs	Frequency	Percent
1	Carcinogenic	134	25
2	Cardiovascular diseases	96	18
3	Dental caries	305	56
4	Poisoning	78	14
5	Diabetes	328	60
6	Habitual addiction	253	47
7	Hypertension	116	21
8	Gastritis	261	48

**Figure 4:**
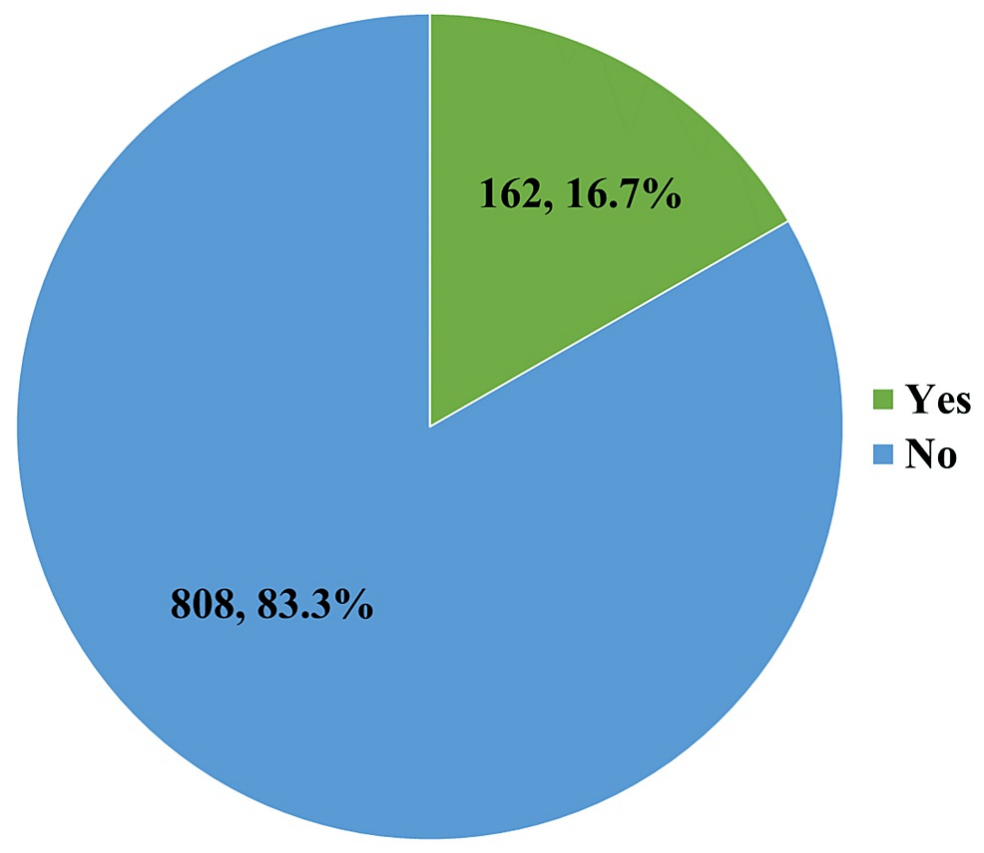
Distribution of the study participants according to their attempt at quitting the consumption of SSBs (n = 970) SSBs: sugar-sweetened beverages

In Figure 4, we describe the participants of the study based on their efforts to cease using SSBs. Only 16.7% of the study participants attempted to break their SSB consumption.

Table 7 depicts the relationship between the typical characteristics of the study group and the consumption of SSBs. According to the chi-square test, characteristics like BMI (overweight), higher economic status, alcohol consumption habit, rural residence, and religion other than Hinduism were statistically associated with SSB intake.

**Table 7:** Association between the risk factors and consumption of SSBs among the study participants (n = 1007) *Fisher's exact test value SSBs: sugar-sweetened beverages

S.no	Risk factors			Sugar-Sweetened drinks consumption		Chi-square value	P-Value
				Yes	No		
1	BMI category	Underweight	N	23	1	11.231*	0.003
			%	95.80%	4.20%		
		Normal	N	342	23		
			%	93.70%	6.30%		
		Overweight	N	605	13		
			%	97.90%	2.10%		
2	Socio-economic status according to Modified P.G. Prasad scale	Class 1	N	637	9	32.855^*^	< 0.001
			%	98.60%	1.40%		
		Class 2	N	186	15		
			%	92.50%	7.50%		
		Class 3	N	79	5		
			%	94.00%	6.00%		
		Class 4	N	61	5		
			%	92.40%	7.60%		
		Class 5	N	7	3		
			%	70.00%	30.00%		
3	Smoking	Yes	N	112	1	2.798^*^	0.112
			%	99.10%	0.90%		
		No	N	858	36		
			%	96.00%	4.00%		
4	Alcohol	Yes	N	191	2	4.695	0.03
			%	99.00%	1.00%		
		No	N	779	35		
			%	95.70%	4.30%		
5	Betel nut chewing	Yes	N	24	0	1.938^*^	1
			%	100.00%	0.00%		
		No	N	946	37		
			%	96.20%	3.80%		
6	Gender	Female	N	369	17	0.942	0.332
			%	95.60%	4.40%		
		Male	N	601	20		
			%	96.80%	3.20%		
7	Residence	Rural / Village	N	428	9	5.688	0.017
			%	97.90%	2.10%		
		Urban / City	N	542	28		
			%	95.10%	4.90%		
8	Education	Graduate	N	608	21	3.636^*^	0.547
			%	96.70%	3.30%		
		High school	N	76	3		
			%	96.20%	3.80%		
		No education	N	29	2		
			%	93.50%	6.50%		
		Post-graduate	N	106	6		
			%	94.60%	5.40%		
		Primary	N	33	2		
			%	94.30%	5.70%		
		Secondary	N	118	3		
			%	97.50%	2.50%		
9	Occupation	Office worker	N	194	8	3.236^*^	0.851
			%	96.00%	4.00%		
		Professional	N	292	8		
			%	97.30%	2.70%		
		Retired	N	21	1		
			%	95.50%	4.50%		
		Self-employed	N	92	4		
			%	95.80%	4.20%		
		Skilled labor	N	107	3		
			%	97.30%	2.70%		
		Student	N	127	7		
			%	94.80%	5.20%		
		Unemployed	N	96	4		
			%	96.00%	4.00%		
		Unskilled labor	N	41	2		
			%	95.30%	4.70%		
10	Marital status	Divorced	N	8	0	2.860^*^	0.239
			%	100.00%	0.00%		
		Married	N	593	18		
			%	97.10%	2.90%		
		Unmarried	N	369	19		
			%	95.10%	4.90%		
11	Religion	Christian	N	94	0	7.518^*^	0.047
			%	100.00%	0.00%		
		Hindu	N	816	37		
			%	95.70%	4.30%		
		Muslim	N	57	0		
			%	100.00%	0.00%		
		Others	N	3	0		
			%	100.00%	0.00%		

## Discussion

We conducted this study with a sample size of 1007 to explore the factors associated with the habit of consuming sugar-sweetened drinks. The prevalence of consuming sugar-sweetened beverages among the participants of our study was determined to be 96.3%. We observed that around 48.8 percent of the study group had consumed sugar-sweetened beverages for more than a decade. A cohort study conducted in the United States by Ren et al. supports our findings that only 12% of study participants did not consume carbonated beverages [25].

In this study, we found that regular use of sugary beverages was associated with being overweight, having a high socioeconomic position, and living in a rural location. Comparable to our findings, a study conducted in Delhi in 2020 by Mathur et al. showed a statistically significant relationship between socioeconomic status and the use of sugar-sweetened beverages. However, the same publication contradicts our conclusion by saying that urban residents had an association with sugary drink consumption. They also discovered a statistically significant association between the male gender and intake of sugar-sweetened beverages, whereas we observed no such relationship [2]. In a systematic review of 11 studies by a different researcher, we found the same results. They concluded that the consumption of sugar-sweetened beverages was associated with obesity [9].

A systematic review conducted by Cheungpastiporn et al. in 2015 in the United States found a statistically significant relationship between the intake of carbonated beverages and hypertension [12]. However, our research found no significant association between them. This discussion requires additional research. Another study, conducted by Goyal et al. in Gujarat among 1209 schoolchildren, found a link between obesity and carbonated beverage consumption [26]. There was a statistically significant association between sugar-sweetened beverages and obesity according to a study conducted by Kumar et al. in India on 500 school-aged children to examine obesity among them [27]. So, there was a strong link between being overweight and drinking sugary water among both teens and young adults.

In 2019, Shetty et al. did research to find out what effect sugary drinks have on blood pressure. They discovered a statistically significant relationship between soft drink consumption and an increase in individuals' mean arterial pressure [28]. Even though we didn't measure the blood pressure of the people in our study, the above conclusion opens a new line of inquiry.

Besides what we have already said, the current study found that about 21% of the people who took part in the study said that drinking sugary drinks made them feel bad, with stomach problems being the most common. In 2016, Ghoshal et al. conducted a community-based study among the general population with functional gastric disorders and found a statistically significant association between sugar-sweetened beverages and the occurrence of irritable bowel syndrome [29]. The findings of this study supported these results. Our survey also revealed that just 16% of participants attempted to give up sugar-sweetened beverages. Nearly 33.4% of the participants had peer influence on SSB consumption, and adults share the same desire to be accepted by the group, particularly their peers. This peer group has a significant probability of affecting adolescents' consumption, as well. We need more research to further explore these findings.

Limitations

The current study used convenient sampling because of limited resources. Probability sampling may produce more generalizable results. We used a semi-structured questionnaire with subjective responses rather than scales to evaluate the detrimental impacts of sugar-sweetened beverages. This may cause informational or recall bias. As this was a cross-sectional study, the association was found to lack a temporal association (e.g., an overweight association with sugar-sweetened drink consumption).

## Conclusions

The prevalence of SSB use among the current study population is exceptionally high. Half of the population has consumed SSBs between 100 and 200 ml for over 100 years. Taste and peer pressure are the primary reasons for facilitating SSBs, whereas the media has a minor impact. Most of the population began consuming SSBs, mostly during vacations and at parties. About one-fifth of the population experiences negative consequences after ingesting SSBs while only half of the population is aware of the contents of SSBs. Likewise, just 50% of the population is aware of the long-term implications of SSBs. A tiny percentage of the population attempted to stop using SSBs. Being overweight, having a high socioeconomic class, and dwelling in a rural location are risk factors related to the consumption of SSBs. Limiting these factors can aid in reducing their consumption, thereby reducing the burden of non-communicable diseases. Our findings highlight the difficulties of developing effective interventions that account for the variability of beverage consumption determinants across sociodemographic characteristics.

There is a need to educate the public about the short- and long-term negative effects of consuming SSBs. Government and non-government entities must work together to generate public behavior and change communication. The effects of SSB use are not commonly explored in research studies and require further investigation.
